# Discovery of Novel Diterpenoids from *Sinularia arborea*

**DOI:** 10.3390/md12010385

**Published:** 2014-01-17

**Authors:** Kuan-Hua Chen, Chang-Feng Dai, Tsong-Long Hwang, Chun-Yu Chen, Jan-Jung Li, Jih-Jung Chen, Yang-Chang Wu, Jyh-Horng Sheu, Wei-Hsien Wang, Ping-Jyun Sung

**Affiliations:** 1 Graduate Institute of Marine Biotechnology, Department of Life Science and Institute of Biotechnology, National Dong Hwa University, Pingtung 944, Taiwan; E-Mail: asdfgh0213@gmail.com; 2 National Museum of Marine Biology and Aquarium, Pingtung 944, Taiwan; E-Mails: jj@nmmba.gov.tw (J.-J.L.); whw@nmmba.gov.tw (W.-H.W.); 3 Institute of Oceanography, National Taiwan University, Taipei 106, Taiwan; E-Mail: corallab@ntu.edu.tw; 4 Graduate Institute of Natural Products, Chang Gung University, Taoyuan 333, Taiwan; E-Mail: htl@mail.cgu.edu.tw; 5 Department of Anesthesiology, Chang Gung Memorial Hospital, Taoyuan 333, Taiwan; E-Mail: rainywoo2000@yahoo.com.tw; 6 Graduate Institute of Clinical Medical Sciences, Chang Gung University, Taoyuan 333, Taiwan; 7 Department of Pharmacy and Graduate Institute of Pharmaceutical Technology, Tajen University, Pingtung 907, Taiwan; E-Mail: jjchen@mail.tajen.edu.tw; 8 School of Pharmacy, College of Pharmacy, China Medical University, Taichung 404, Taiwan; E-Mail: yachwu@mail.cmu.edu.tw; 9 Chinese Medicine Research and Development Center, China Medical University Hospital, Taichung 404, Taiwan; 10 Center for Molecular Medicine, China Medical University Hospital, Taichung 404, Taiwan; 11 Department of Marine Biotechnology and Resources and Division of Marine Biotechnology, Asia-Pacific Ocean Research Center, National Sun Yat-sen University, Kaohsiung 804, Taiwan; E-Mail: sheu@mail.nsysu.edu.tw; 12 Doctoral Degree Program in Marine Biotechnology, National Sun Yat-sen University and Academia Sinica, Kaohsiung 804, Taiwan; 13 Graduate Institute of Natural Products, Kaohsiung Medical University, Kaohsiung 807, Taiwan

**Keywords:** sinularbol, sinularborane, diterpenoid, *Sinularia arborea*, superoxide anion

## Abstract

Two novel diterpenoids, sinularbols A (**1**) and B (**2**), which were found to possess a new carbon skeleton were isolated from the soft coral *Sinularia arborea*. The structures of compounds **1** and **2** were elucidated by spectroscopic methods and **2** displayed a moderately inhibitory effect on the generation of superoxide anion by human neutrophils.

## 1. Introduction

**Scheme 1:**
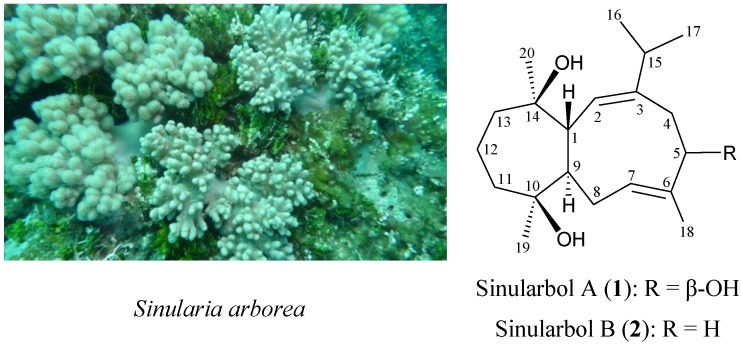
The soft coral *Sinularia arborea* and the structures of sinularbols A (**1**) and B (**2**).

Previous studies on the chemical constituents of octocorals, belonging to the genus *Sinularia,* have led to the isolation of a number of interesting secondary metabolites and some of these were found to possess interesting bioactivities [1,2]. In a previous study on the chemical constituents of the soft coral *Sinularia arborea* (phylum Cnidaria, class Anthozoa, order Alcyonacea, family Alcyoniidae) (Scheme 1) [3], collected off the waters of Taiwan, two 13-hydroxycembrane diterpenoids, arbolides A and B along with a trihydroxysteroid, crassarosterol A, had been isolated [4]. In the continuing studies on this interesting species, two novel diterpenoids, sinularbols A (**1**) (Scheme 1 and Supplementary Figures S1–S8) and B (**2**) (Scheme 1 and Supplementary Figures S9–S16), which were found to possess a novel sinularborane-type carbon skeleton (3,9-cyclized cembranoid), were obtained (Scheme 1). In this paper, we describe the isolation, structure determination and bioactivity of diterpenoids **1** and **2**.

## 2. Results and Discussion

Sinularbol A (**1**) was isolated as colorless oil and its molecular formula was established as C_20_H_34_O_3_ from a sodiated ion at *m/z* 345 in the ESIMS and further supported by the HRESIMS at *m/z* 345.2404 (calcd. for C_20_H_34_O_3_Na, 345.2406) and implying four degrees of unsaturation. The presence of hydroxy groups in **1** was evidenced by a broad IR absorption at υ_max_ 3435 cm^–1^. The ^13^C spectrum of **1** showed 20 carbon signals (Table 1), which were assigned by the assistance of DEPT spectrum to five methyls, five sp^3^ methylenes, four sp^3^ methines (including an oxymethine), two sp^2^ methines, two sp^3^ oxygenated quaternary carbons and two sp^2^ quaternary carbons. From the ^13^C and ^1^H NMR data (Table 1), two trisubstituted olefins were deduced from the signals at δ_C_ 146.7 (C), 137.4 (C), 127.7 (CH) and 125.5 (CH), and confirmed by two olefin proton signals at δ_H_ 5.58 (1H, dd, *J* = 6.4, 6.4 Hz) and 5.20 (1H, d, *J* = 9.6 Hz) in the ^1^H NMR spectrum. Comparison of the ^13^C NMR and DEPT spectra with the molecular formula indicated that there must be three exchangeable protons, requiring the presence of three hydroxy groups. From the above data, two degrees of unsaturation were accounted for and **1** must be a diterpenoid derivative with two rings.

**Table 1:** ^1^H (400 MHz, CDCl_3_) and ^13^C (100 MHz, CDCl_3_) NMR data, ^1^H–^1^H COSY and HMBC correlations for sinularbol A (**1**).

Position	δ_H_ (*J* in Hz)	δ_C_, Multiple		^1^H–^1^H COSY	HMBC
1	2.45 dd (11.2, 9.6)	49.9	CH	H-2, H-9	C-2, -3, -9, -10, -14
2	5.20 d (9.6)	125.5	CH	H-1	C-1, -4, -9, -15
3		146.7	C		
4	2.12 dd (12.8, 2.8)	38.3	CH_2_	H-5	C-2, -3, -5, -6, -15
	2.94 dd (12.8, 11.2)				
5	4.03 ddd (11.2, 2.8, 2.8)	76.0	CH	H_2_-4, OH-5	n.o. ^a^
6		137.4	C		
7	5.58 dd (6.4, 6.4)	127.7	CH	H_2_-8	C-5, -18
8	2.16 m; 2.27 m	22.4	CH_2_	H-7, H-9	C-7, -10
9	1.97 m	56.6	CH	H-1, H_2_-8	n.o.
10		74.7	C		
11	1.62 m; 1.98 m	33.9	CH_2_	H_2_-12	C-9, -10
12	1.34 m; 1.66 m	23.4	CH_2_	H_2_-11, H_2_-13	n.o.
13	1.68–1.75 m	39.5	CH_2_	H_2_-12	C-1, -12, -14
14		81.5	C		
15	2.30 m	34.6	CH	H_3_-16, H_3_-17	C-2, -3, -4, -16, -17
16	1.09 d (6.8)	24.1	CH_3_	H-15	C-15, -17
17	1.09 d (6.8)	24.2	CH_3_	H-15	C-15, -17
18	1.78 s	12.7	CH_3_		C-5, -6, -7
19	1.16 s	31.3	CH_3_		C-9, -10, -11
20	1.09 s	22.2	CH_3_		C-1, -13, -14
OH-5	1.53 d (2.8)			H-5	n.o.
OH-10	2.08 s				C-9, -10, -19
OH-14	2.18 s				C-13, -14

^a^ n.o. is not observed.

From the ^1^H–^1^H COSY spectrum of **1**, the separate spin systems of H_2_-4/H-5 and H-7/H_2_-8/H-9/H-1/H-2 were differentiated. These data, together with the HMBC correlations among H-1/C-2, -3, -9; H-2/C-1, -4, -9; H_2_-4/C-2, -3, -5, -6; H-7/C-5; H-8/C-7, established the connectivity from C-1 to C-9 in the nine-membered ring (Table 1). The 1,4-dimethylcycloheptane ring, which is fused to the nine-membered ring at C-1 and C-9, was elucidated by the HMBC correlations between H-1/C-10, -14; H_2_-8/C-10; H_2_-11/C-9; H_2_-13/C-1; H_3_-19/C-9, -10, -11; H_3_-20/C-1, -13, -14; and OH-10/C-9. The isopropyl group was established by the ^1^H–^1^H COSY correlations between H-15/H_3_-16 (H_3_-17) and this group at C-3 from the HMBC correlations among H-2/C-15; H_2_-4/C-15; and H-15/C-2, -3, -4. A vinyl methyl at C-11 was confirmed by the HMBC correlations between H_3_-18/C-5, -6, -7 and H-7/C-18. The hydroxy proton signal at δ_H_ 1.53 (1H, d, *J* = 2.8 Hz, OH-5) was revealed by its ^1^H–^1^H COSY correlation to δ_H_ 4.03 (1H, d, *J* = 11.2, 2.8, 2.8 Hz, H-5), indicating its attachment to C-5. These data, together with the HMBC correlations between OH-10 (δ_H_ 2.08)/C-9, -10, -19 and OH-14 (δ_H_ 2.18)/C-13, -14, unambiguously established the molecular framework of **1**.

**Scheme 2:**
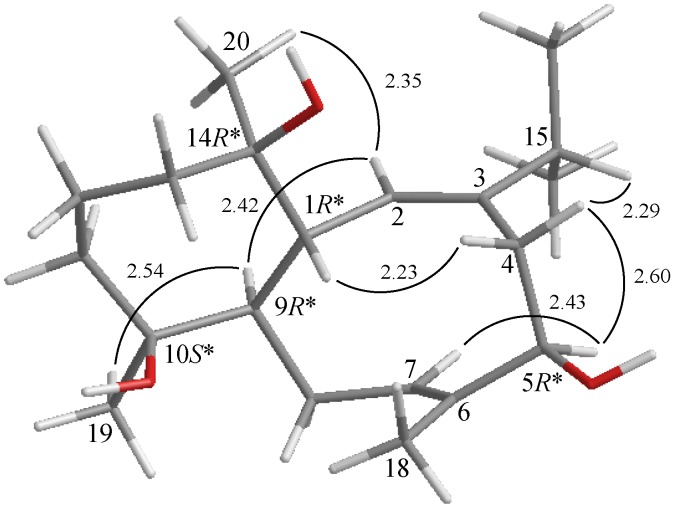
The computer-generated model of **1** using MM2 force field calculations and the calculated distances (Å) between selected protons with key NOESY correlations.

The relative configuration of **1** was elucidated from the interactions observed in a NOESY experiment and was found to be compatible with that of **1** offered by computer modeling (Scheme 2) [5] and that obtained from vicinal proton coupling constant analysis. Because of the β-orientation of H-1, and this proton correlated with one of the methylene protons at C-4 (δ_H_ 2.94), and therefore it was assigned as H-4β and the other C-4 proton (δ_H_ 2.12) as H-4α. H-5 correlated with H-4α, but not with H-4β, and a large coupling constant was recorded between these H-5 and H-4β (*J* = 11.2 Hz), indicated that the 5-hydroxy group was β-oriented. Large coupling constants were detected between H-1/H-2 (*J* = 9.6 Hz) and H-1/H-9 (*J* = 11.2 Hz) and there is no correlation was found between H-1/H-2 and H-1/H-9, indicating that the dihedral angles between H-1/H-2 and H-1/H-9 are approximately 180°, respectively, and H-9 should be α-oriented. This structure was further supported by a correlation between H-2 and H-9. H_3_-20 showed a correlation with H-2, but not with H-1, demonstrating the α-orientation of Me-20. The C-19 methyl showed a correlation with H-9, indicating that the hydroxy group at C-10 was β-oriented. Correlations between H-5/H-7 and H-4α/H-15, as well as the lack of correlation between H-7/H_3_-18 and H-2/H_2_-4, reflected the *E* geometry of the double bonds at C-2/3 and C-6/7. Based on the above findings, the structure of **1** was elucidated and the chiral carbons for **1** were assigned as 1R*, 5R*, 9R*, 10S* and 14R*.

The new sinularborane **2** (sinularbol B) was isolated as colorless oil and its molecular formula C_20_H_34_O_2_ was established by HRESIMS (*m/z* 329.2453, calcd. for C_20_H_34_O_2_Na, 329.2456). The IR spectrum of **2** showed a broad band at 3447 cm^–1^, consistent with the presence of hydroxy groups. It was found that the ^1^H and ^13^C NMR data of **2** were similar to those of **1**, except that the signals corresponding to the C-5, a hydroxy-bearing oxymethine group in **1** (δ_H_ 4.03, 1H, ddd, *J* = 11.2, 2.8, 2.8 Hz; δ_C_ 76.0, CH) were replaced by a methylene group in **2** (δ_H_ 1.88, 1H, m; 2.22, 1H, m; δ_C_ 35.9, CH_2_) (Table 2). The ^1^H–^1^H COSY and HMBC correlations observed fully supported the locations of functional groups (Table 2), and hence sinularbol B (**2**) was assigned as the structure **2** with the same relative stereochemistry as in sinularbol A (**1**) because for the chiral carbons that **2** had in common with **1** and the relative configurations of **2** were assigned as 1R*, 9R*, 10S* and 14R*. The ^1^H and ^13^C NMR chemical shifts and proton coupling constants match well, and are further supported by a NOESY experiment.

**Table 2:** ^1^H (400 MHz, CDCl_3_) and ^13^C (100 MHz, CDCl_3_) NMR data, ^1^H–^1^H COSY and HMBC correlations for sinularbol B (**2**).

Position	δ_H_ (*J* in Hz)	δ_C_, Multiple		^1^H–^1^H COSY	HMBC
1	2.54 dd (11.2, 9.6)	50.2	CH	H-2, H-9	C-2, -3, -9, -10, -14, -20
2	5.19 dd (9.6)	124.0	CH	H-1	C-1, -4, -9, -15
3		149.0	C		
4	1.99 m	29.5	CH_2_	H_2_-5	C-2, -3, -5, -6, -15
	2.72 ddd (13.2, 13.2, 2.8)				
5	1.88 m; 2.22 m	35.9	CH_2_	H_2_-4	C-3, -4, -6, -7, -18
6		134.8	C		
7	5.32 dd (5.6, 5.6)	127.7	CH	H_2_-8	n.o. ^a^
8	2.08 m; 2.30 m	22.8	CH_2_	H-7, H-9	C-7, -10
9	1.97 m	56.6	CH	H-1, H_2_-8	n.o.
10		74.7	C		
11	1.59 m; 1.88 m	34.1	CH_2_	H_2_-12	C-9, -12, -19
12	1.35 m; 1.66 m	23.3	CH_2_	H_2_-11, H_2_-13	C-10
13	1.68–1.77 m	39.2	CH_2_	H_2_-12	C-1, -14, -20
14		81.5	C		
15	2.27 m	33.2	CH	H_3_-16, H_3_-17	C-2, -3, -16, -17
16	1.06 d (6.8)	24.1	CH_3_	H-15	C-3, -15, -17
17	1.09 d (6.8)	24.4	CH_3_	H-15	C-3, -15, -16
18	1.70 s	18.2	CH_3_		C-5, -6, -7
19	1.51 s	31.6	CH_3_		C-9, -10, -11
20	1.08 s	22.1	CH_3_		C-1, -13, -14

^a^ n.o. = not observed.

The *in vitro* anti-inflammatory effects of compounds **1** and **2** were examined and **2** displayed a moderately inhibitory effect on the generation of superoxide anion by human neutrophils (Table 3) [6].

**Table 3:** Inhibitory effects of diterpenoids **1** and **2** on the generation of superoxide anion and the release of elastase by human neutrophils in response to FMLP/CB.

Compound	Superoxide anion	Elastase release
	Inh%	Inh%
**1**	7.37 ± 1.98 *	11.71 ± 1.35 ***
**2**	23.94 ± 6.35 *	6.54 ± 3.54

Percentage of inhibition (Inh%) at a concentration of 10 μg/mL. The results are presented as mean ± S.E.M. (*n* = 3). * *p* < 0.05; *** *p* < 0.001, compared with the control value.

## 3. Experimental Section

### 3.1. General Experimental Procedures

Optical rotations were measured at a Jasco P-1010 digital polarimeter (Japan Spectroscopic Corporation, Tokyo, Japan). Infrared spectra were recorded on a Varian Diglab FTS 1000 FT-IR spectrometer (Varian Inc., Palo Alto, CA, USA); peaks are reported in cm^–1^. NMR spectra were recorded on a Varian Mercury Plus 400 NMR spectrometer (Varian Inc., Palo Alto, CA, USA) using the residual CHCl_3_ signal (δ_H_ 7.26 ppm) as the internal standard for ^1^H NMR and CDCl_3_ (δ_C_ 77.1 ppm) for ^13^C NMR. Coupling constants (*J*) are given in Hz. ESIMS and HRESIMS were recorded using a Bruker APEX II FT mass spectrometer (Bruker, Bremen, Germany). Column chromatography was performed on silica gel (230–400 mesh, Merck, Darmstadt, Germany). TLC was carried out on precoated Kieselgel 60 F_254_ (0.25 mm, Merck); spots were visualized by spraying with 10% H_2_SO_4_ solution followed by heating. The normal phase HPLC (NP-HPLC) was performed using a system comprised of a Hitachi L-7110 pump (Hitachi Ltd. Tokyo, Japan) and a Rheodyne 7725 injection port (Rheodyne LLC. Rohnert Park, CA, USA). A normal phase column (Supelco Ascentis^®^ Si Cat #: 581515-U, 25 cm × 21.2 mm, 5 µm, Sigma-Aldrich. Com. St. Louis, MO, USA) was used for NP-HPLC. The reverse phase HPLC (NP-HPLC) was performed using a system comprised of a Hitachi L-7100 pump (Hitachi Ltd., Tokyo, Japan), a Hitachi L-2455 photodiode array detector (Hitachi Ltd., Tokyo, Japan), a Rheodyne 7725 injection port (Rheodyne LLC., Rohnert Park, CA, USA) and a Varian Polaris 5 C-18-A column (25 cm × 10 mm, 5 μm).

### 3.2. Animal Material

Specimens of the octocoral *Sinularia arborea* were collected by hand using SCUBA equipment off the coast of southern Taiwan on October, 2012, and stored at −20 °C until extraction. A voucher specimen (NMMBA-TWSC-1200X) was deposited in the National Museum of Marine Biology and Aquarium, Taiwan.

### 3.3. Extraction and Isolation

Specimens of the soft coral *Sinularia arborea* (wet weight 1.6 kg, dry weight 576 g) were minced and extracted with ethyl acetate (EtOAc). The EtOAc extract left after removal of the solvent (12.5 g) was separated by silica gel and eluted using a mixture of *n*-hexane/EtOAc in a stepwise fashion from 100:1–pure EtOAc to yield 11 fractions A–K. Fraction I was chromatographed on silica gel and eluted using a mixture of *n*-hexane and acetone (6:1/4:1/2:1/pure acetone) to yield 25 subfractions I1–I25. Fraction I12 was repurified by NP-HPLC, using a mixture of *n*-hexane and acetone (4:1) to yield 46 subfractions I12-1–I12-46. Fraction I12-43 was repurified by NP-HPLC, using a mixture of *n*-hexane and acetone (4:1, flow rate: 1.0 mL/min) to yield **1** (0.7 mg, *t*_R_ = 73 min). Fraction E was separated by NP-HPLC, using a mixture of dichloromethane and methanol (90:1) to yield 26 subfractions E1–E26. E20 was repurified by NP-HPLC, using a mixture of *n*-hexane and acetone (10:1) to yield 9 subfractions E20A–E20I. Fraction E20F was repurified by RP-HPLC, using a mixture of methanol and water (84:16) to yield 14 subfractions E20F1–E20F14. Fraction E20F2 was further separated by RP-HPLC, using a mixture of acetonitrile and water (4:1, flow rate: 1.0 mL/min) to yield **2** (0.9 mg, *t*_R_ = 31 min).

Sinularbol A (**1**): colorless oil; 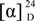 −47 (*c* 0.04, CHCl_3_); IR (neat) υ_max_ 3435 cm^–1^; ^1^H (400 MHz, CDCl_3_) and ^13^C (100 MHz, CDCl_3_) NMR data, see Table 1; ESIMS: *m/z* 345 [M + Na]^+^; HRESIMS: *m/z* 345.2404 (calcd. for C_20_H_34_O_3_Na, 345.2406).

Sinularbol B (**2**): colorless oil; 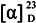 −8 (*c* 0.05, CHCl_3_); IR (neat) υ_max_ 3447 cm^–1^; ^1^H (400 MHz, CDCl_3_) and ^13^C (100 MHz, CDCl_3_) NMR data, see Table 2; ESIMS: *m/z* 327 [M + Na]^+^; HRESIMS: *m/z* 329.2453 (calcd. for C_20_H_34_O_2_Na, 329.2456).

### 3.4. Generation of Superoxide Anions and Release of Elastase by Human Neutrophils

Human neutrophils were obtained by means of dextran sedimentation and Ficoll centrifugation. Measurements of superoxide anion generation and elastase release were carried out according to previously described procedures [7,8,9,10,11,12,13]. Briefly, superoxide anion production was assayed by monitoring the superoxide dismutase-inhibitable reduction of ferricytochrome *c*. Elastase release experiments were performed using MeO-Suc-Ala-Ala-Pro-Valp-nitroanilide as the elastase substrate.

## 4. Conclusions

Octocorals have proven to be rich sources of cyclized cembranoid analogues [14,15,16]. In our continuing investigation on the chemical constituents of marine invertebrates collected off the waters of Taiwan, two novel diterpenoids, sinularbols A (**1**) and B (**2**), which were found to possess a novel sinularborane-type carbon skeleton, were isolated from the soft coral *Sinularia arborea* and **2** was found to show anti-inflammatory activity. It is noteworthy to mention that metabolites **1** and **2** represent the first 3,9-cyclized cembranoid (sinularborane skeleton) derivatives.

## Supplementary Files

Supplementary File 1 Supplementary Information (PDF, 939 KB)
