# Supplementary material for: Discovery of Novel Diterpenoids from Sinularia arborea
Source: Mar Drugs. 2014 Jan 17;12(1):385–93. doi: 10.3390/md12010385 (PMC3917279; doi:10.3390/md12010385)

Supplementary Information

Figure S1. ^{1}H NMR spectrum (400 MHz) of compound **1** in CDCl3.

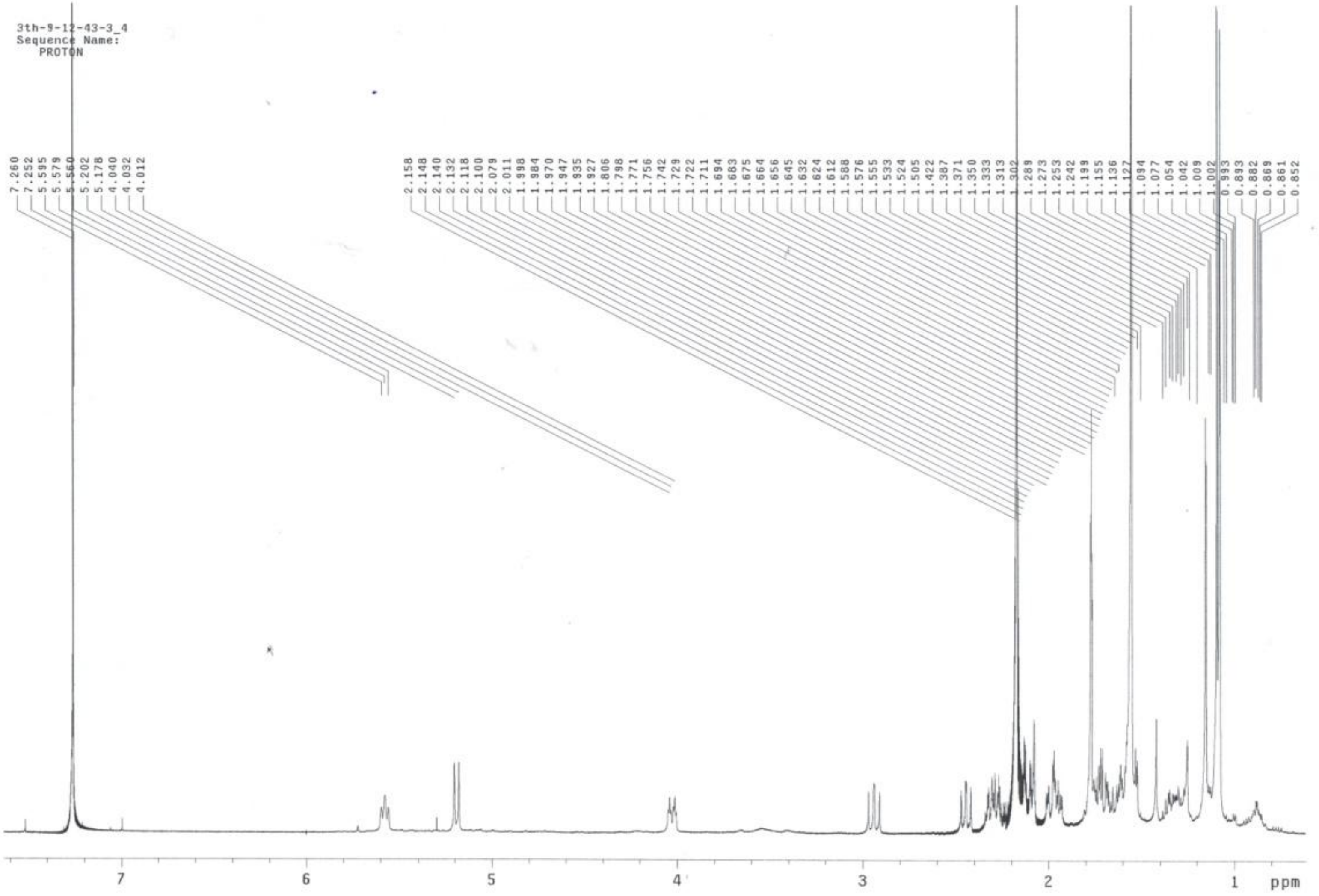

Figure S2. ^{1}H NMR spectrum (400 MHz) of compound **1** in $CDCl_3$.

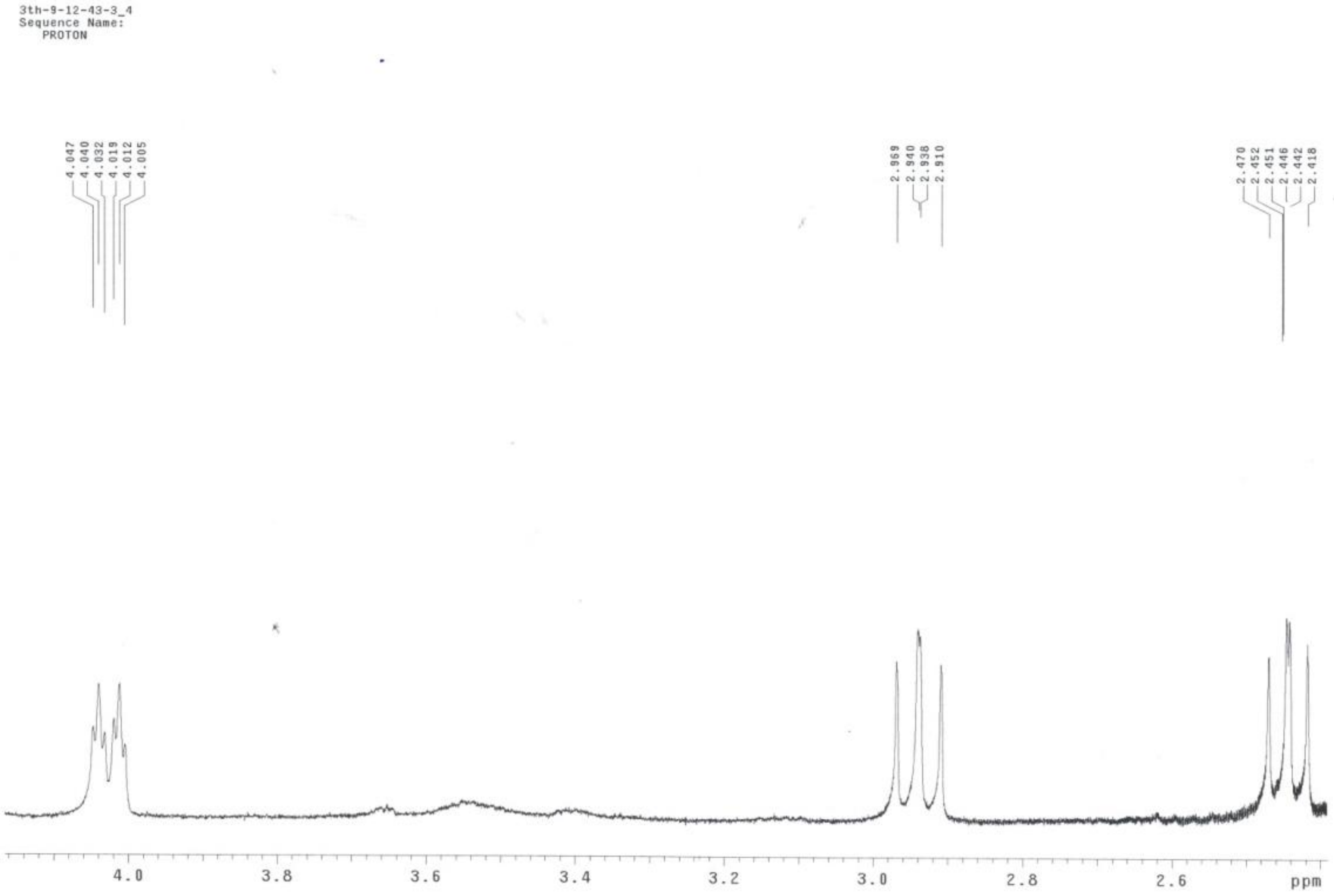

Figure S3. ^{13}C NMR spectrum (100 MHz) of compound **1** in $CDCl_3$.

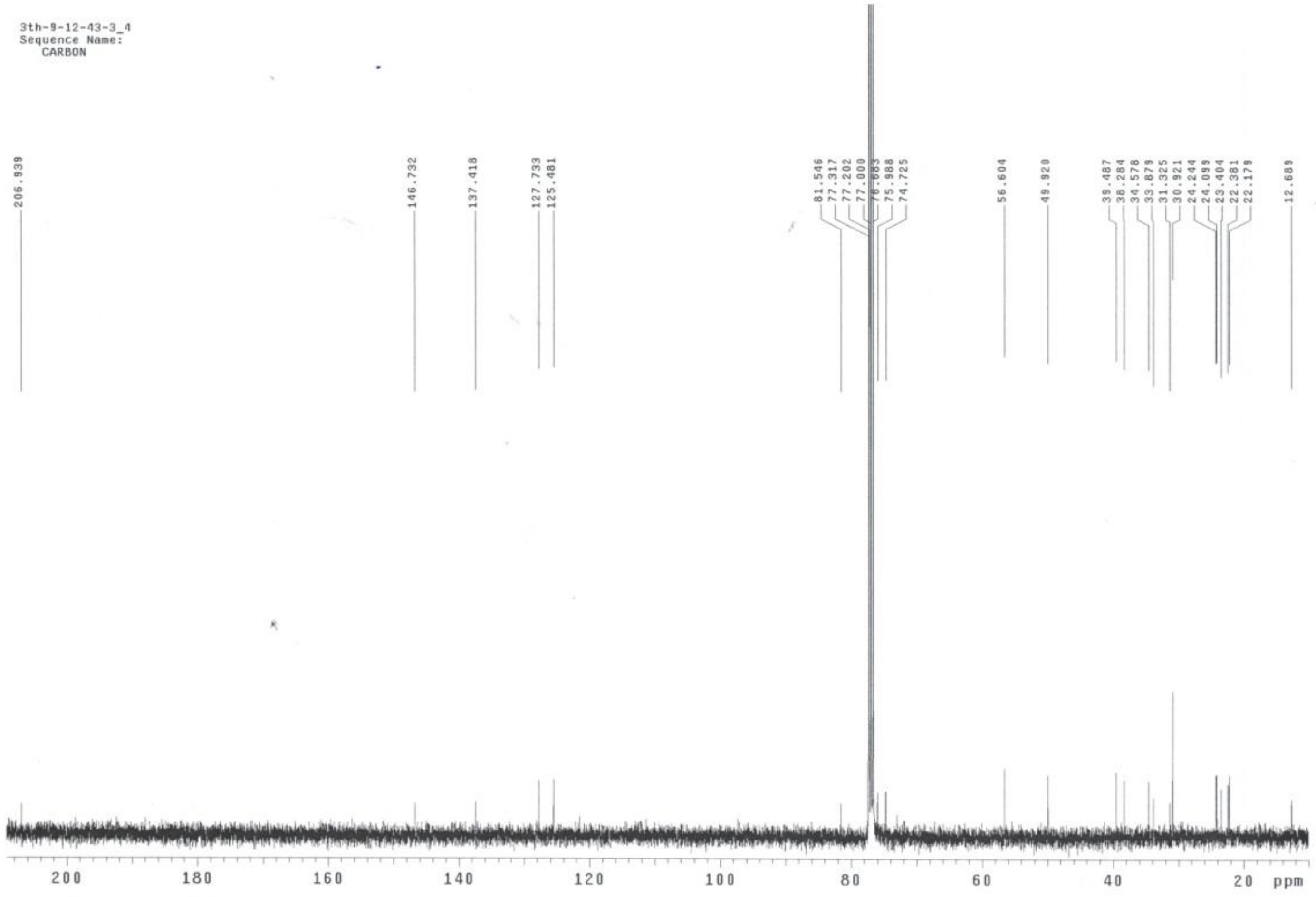

Figure S4. DEPT spectrum (100 MHz) of compound **1** in $CDCl_3$.

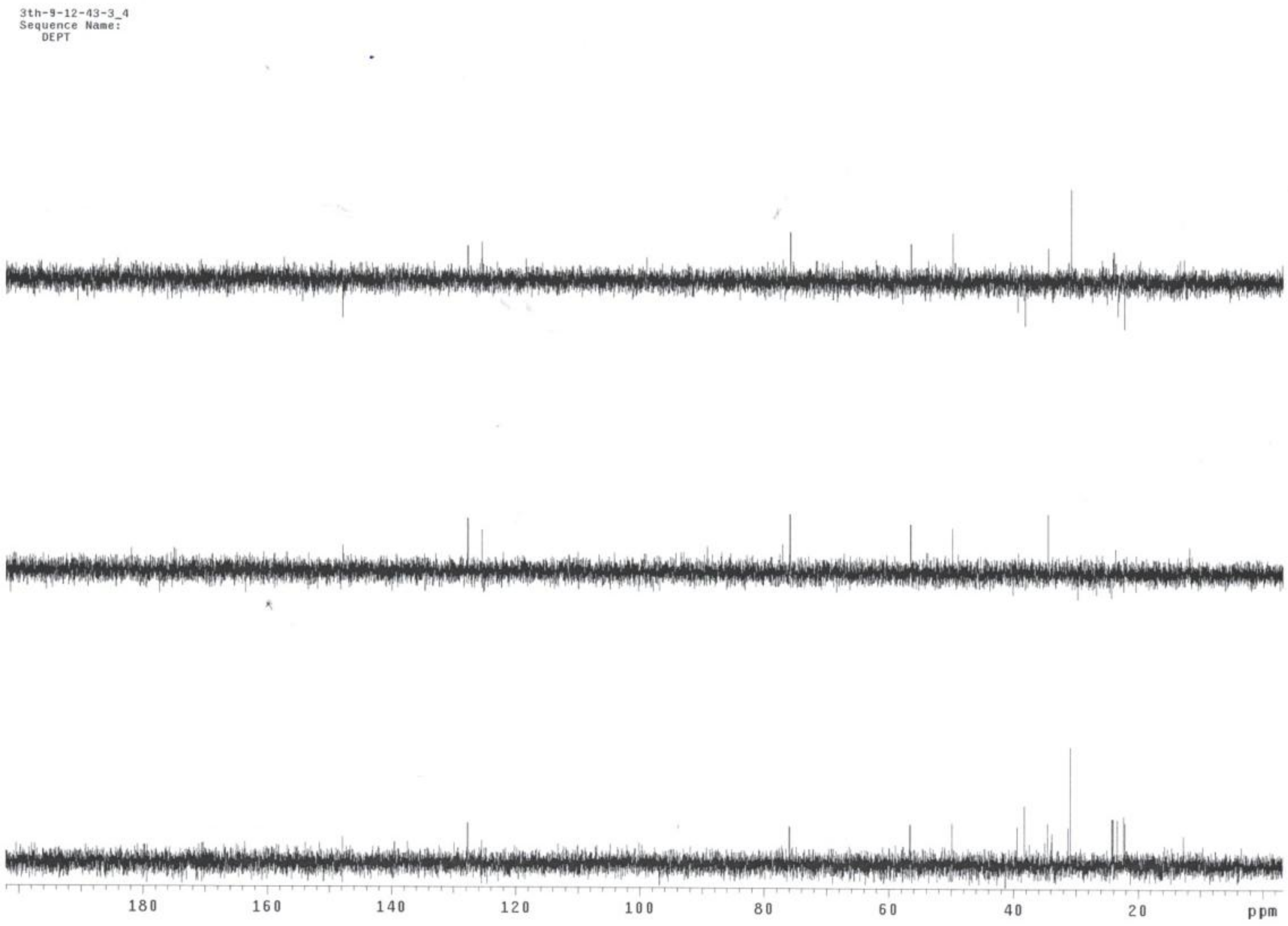

Figure S5. HSQC spectrum (400 MHz) of compound **1** in $CDCl_3$.

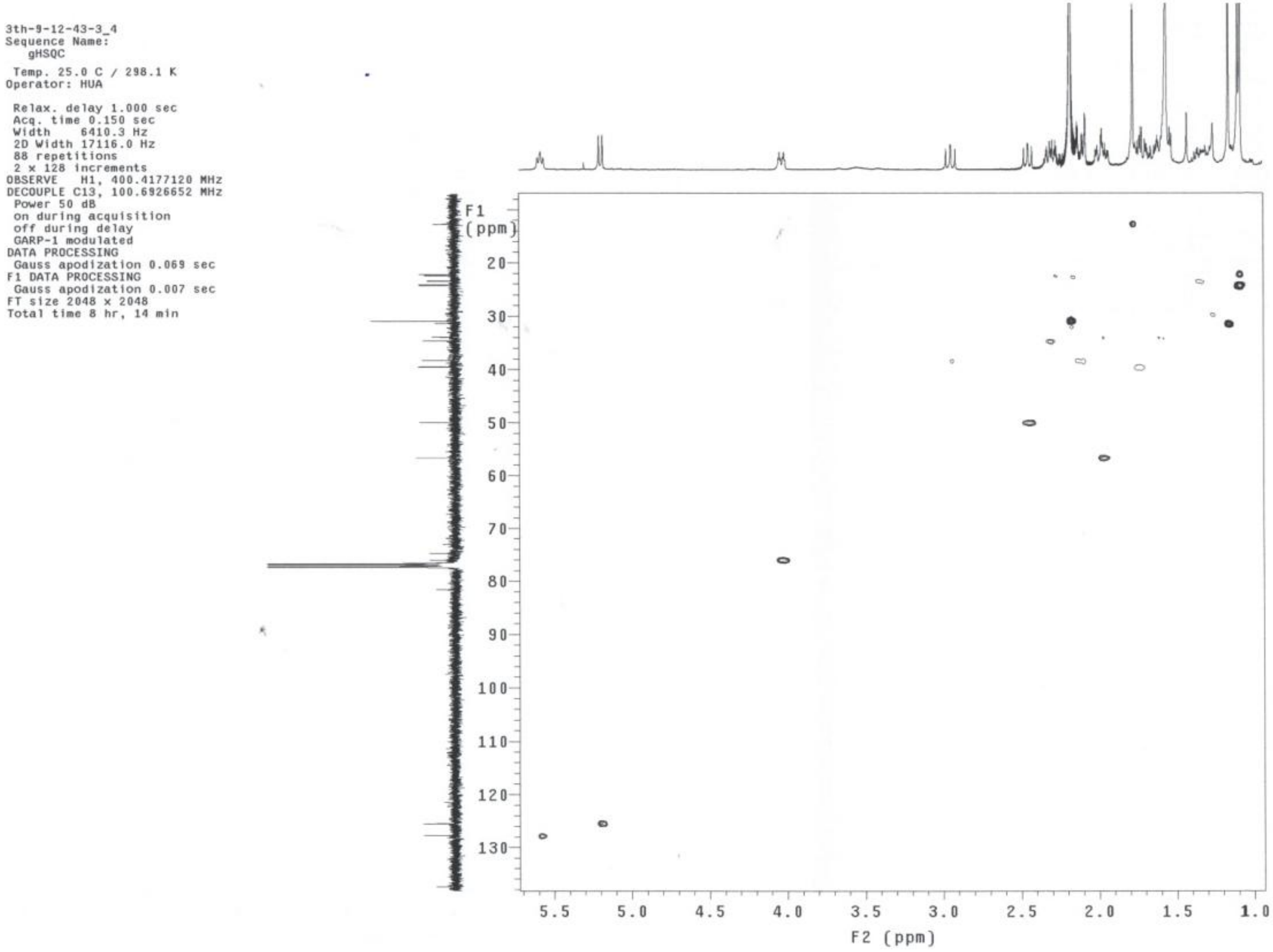

Figure S6. HMBC spectrum (400 MHz) of compound **1** in $CDCl_3$.

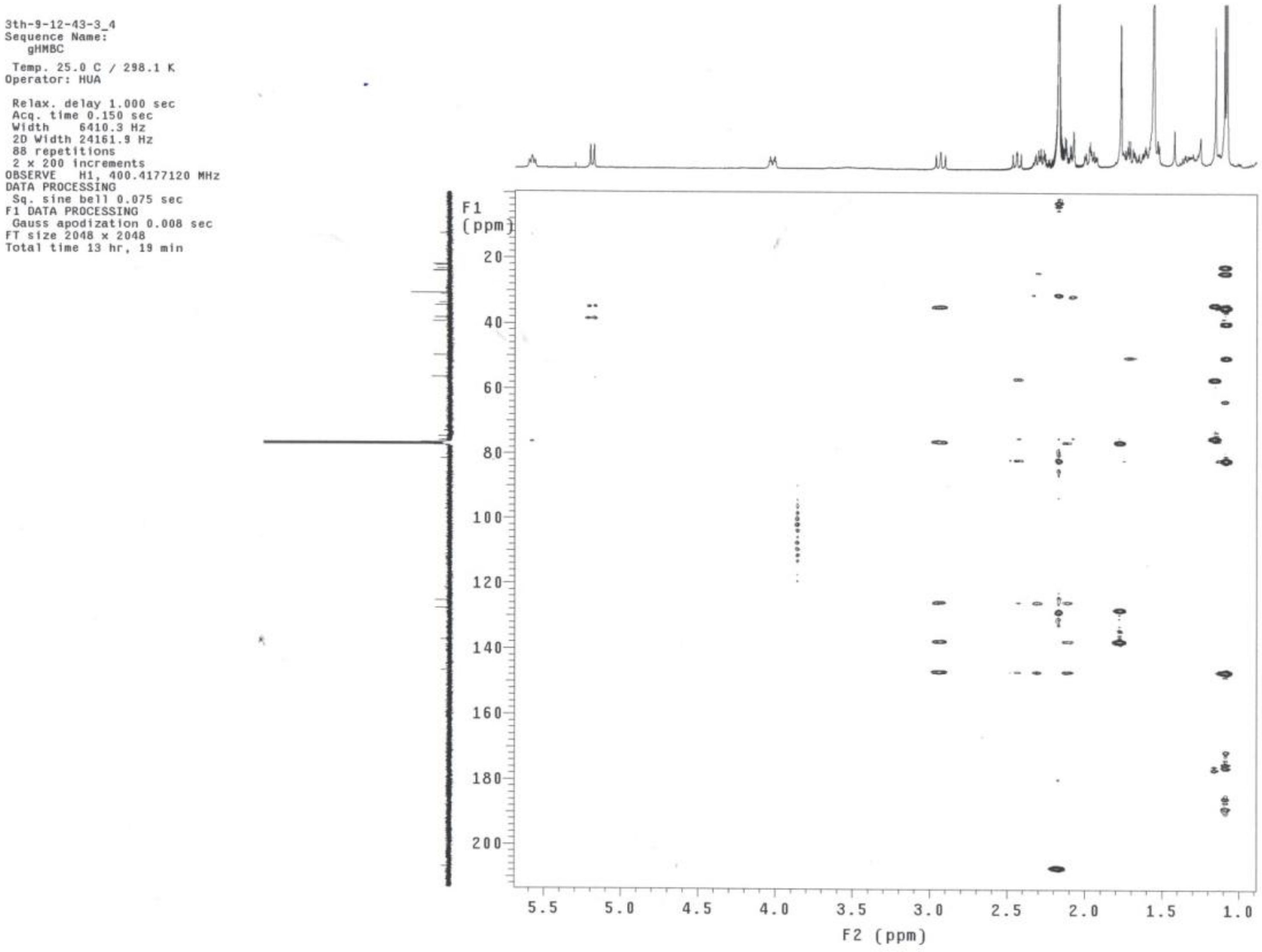

Figure S7. COSY spectrum (400 MHz) of compound **1** in $CDCl_3$.

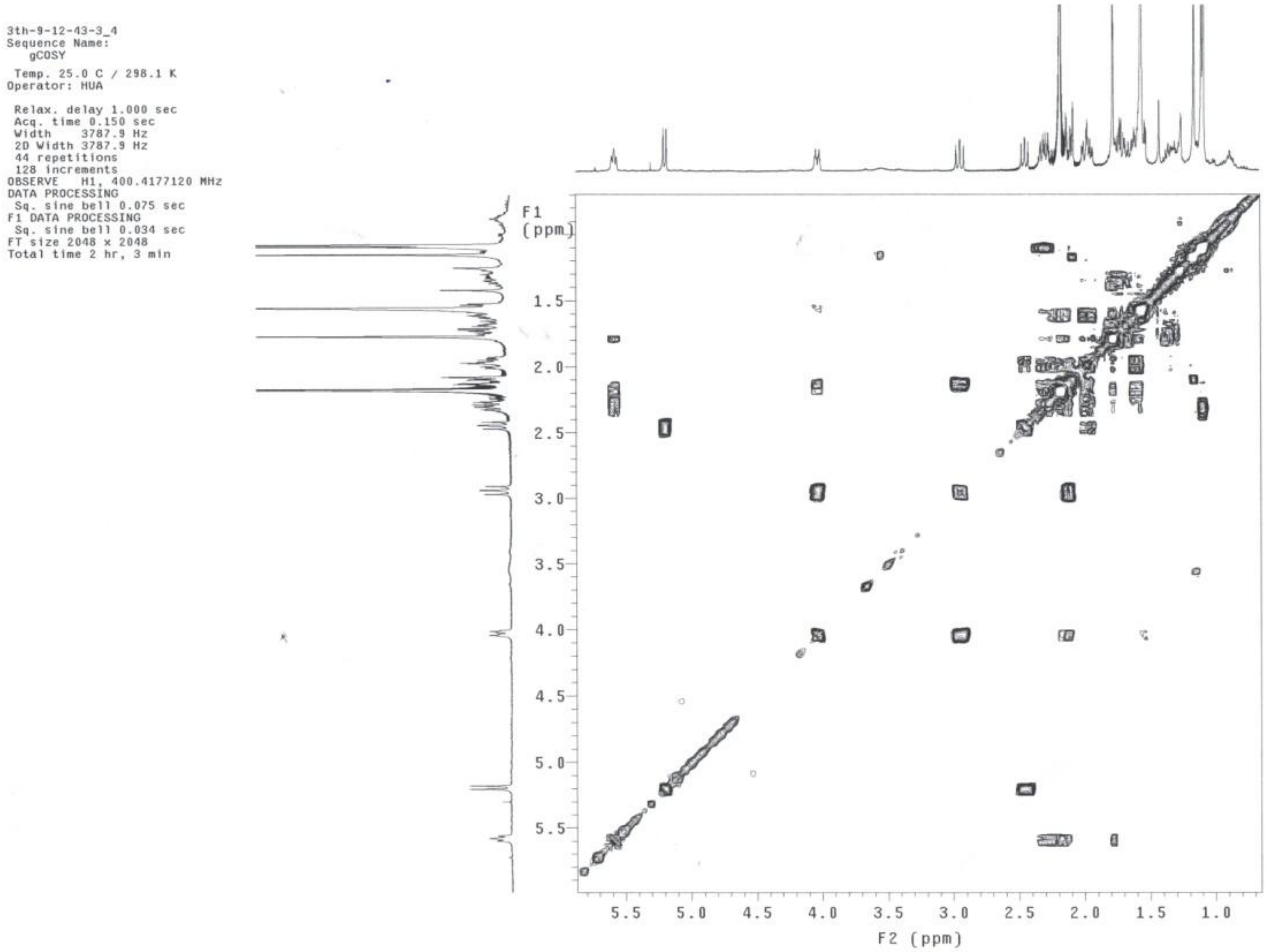

Figure S8. NOESY spectrum (400 MHz) of compound **1** in $CDCl_3$.

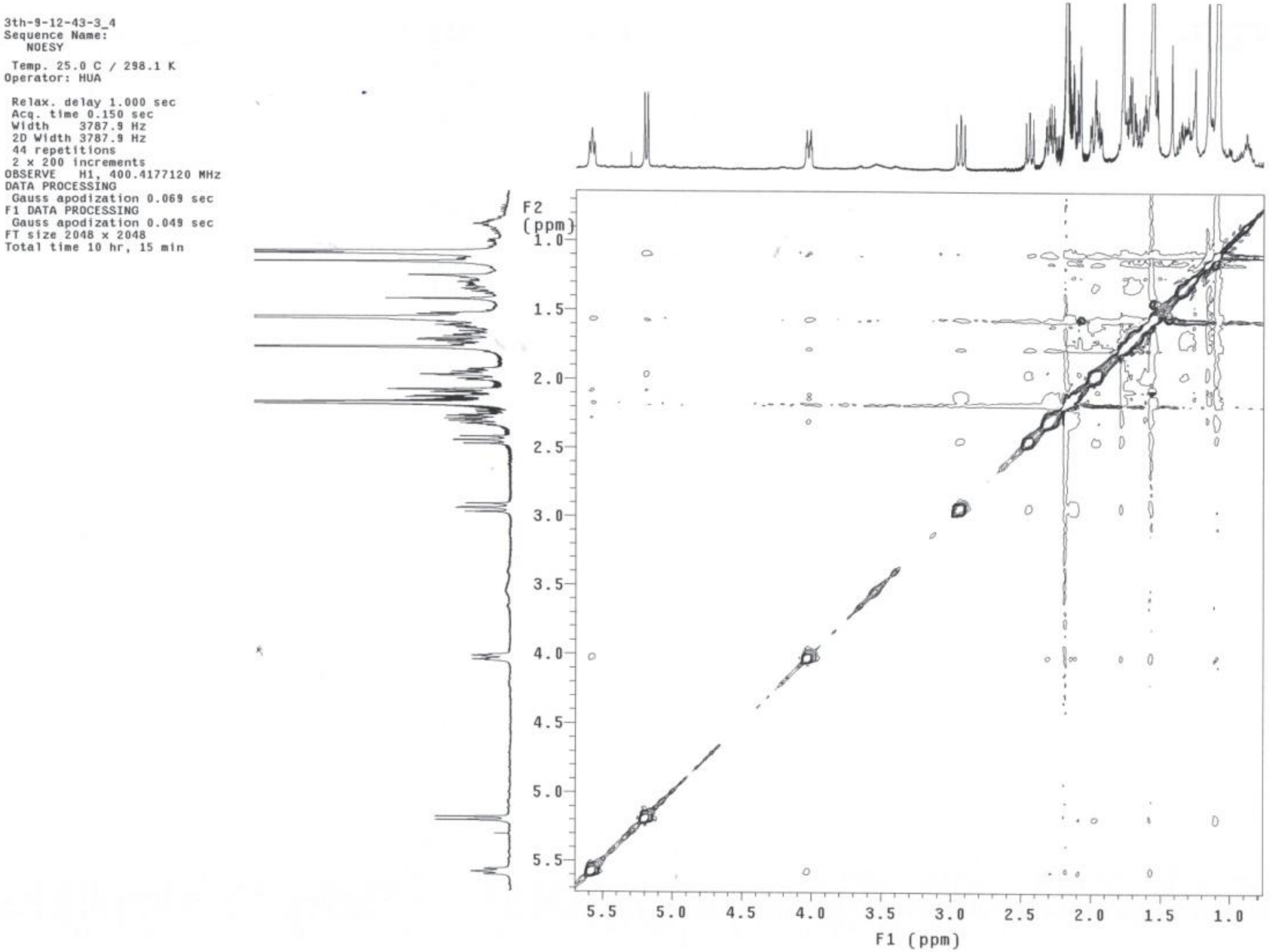

Figure S9. ^{1}H NMR spectrum (400 MHz) of compound **2** in $CDCl_3$.

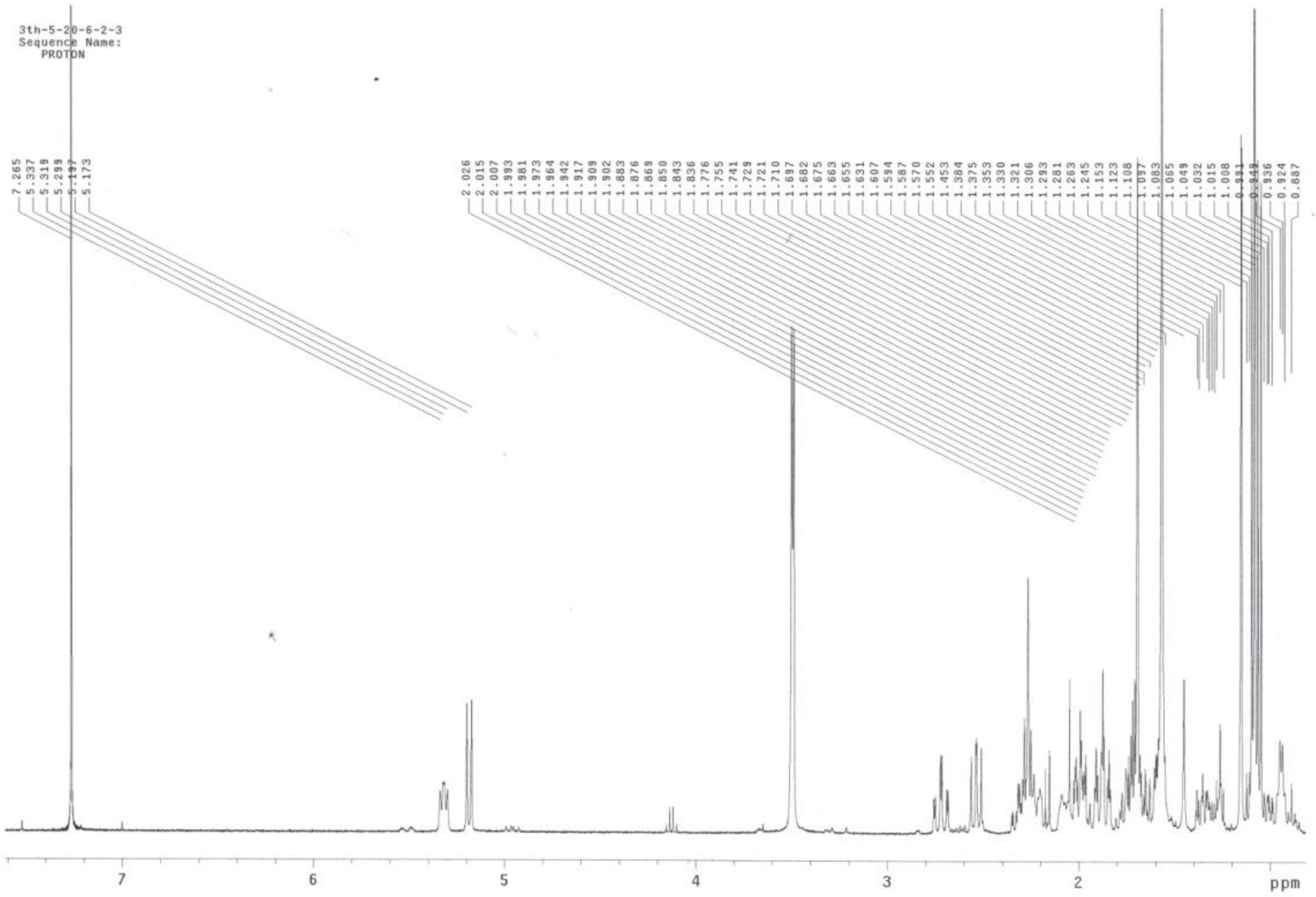

Figure S10. ^{1}H NMR spectrum (400 MHz) of compound **2** in $CDCl_3$.

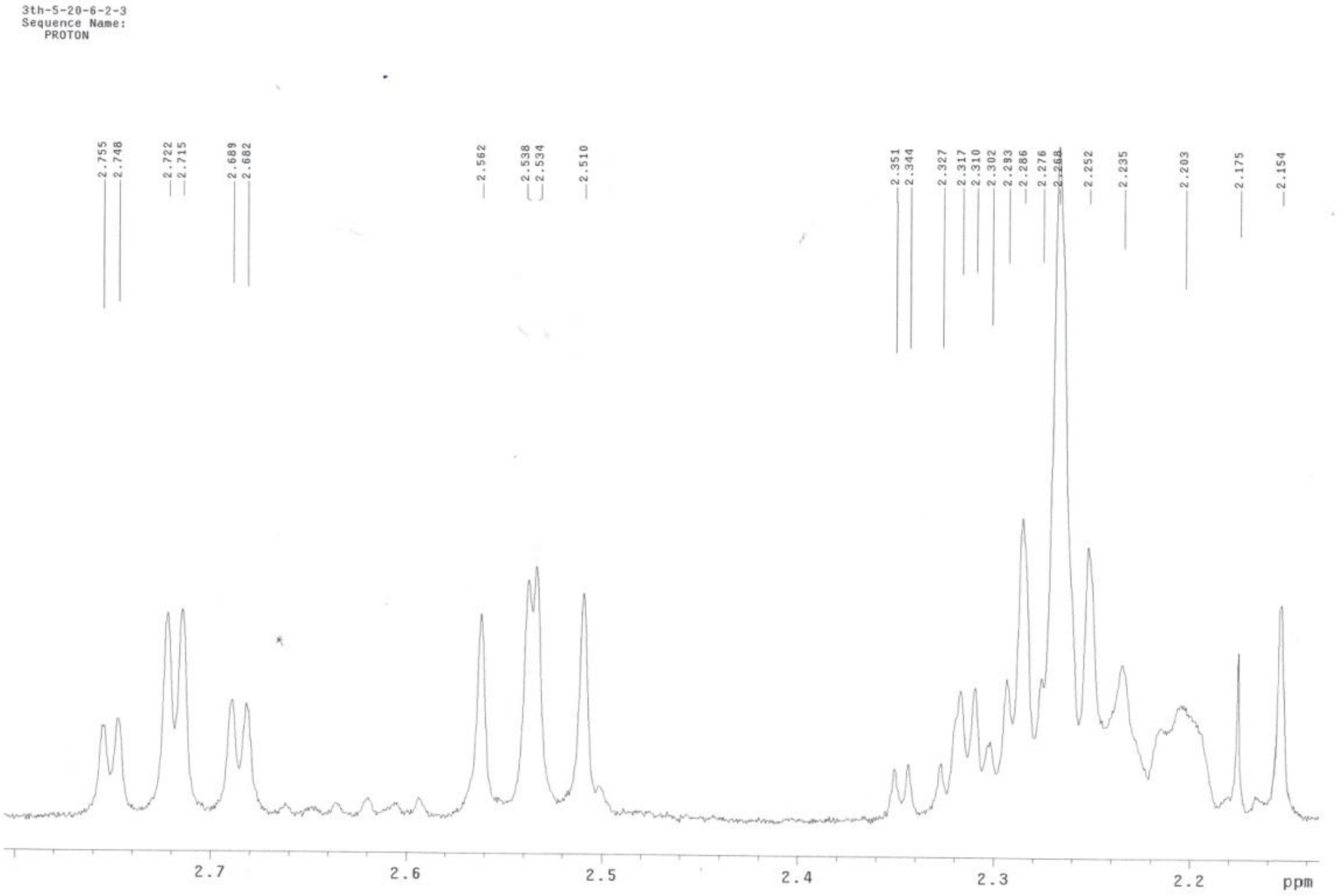

Figure S11. ^{13}C NMR spectrum (100 MHz) of compound **2** in $CDCl_3$.

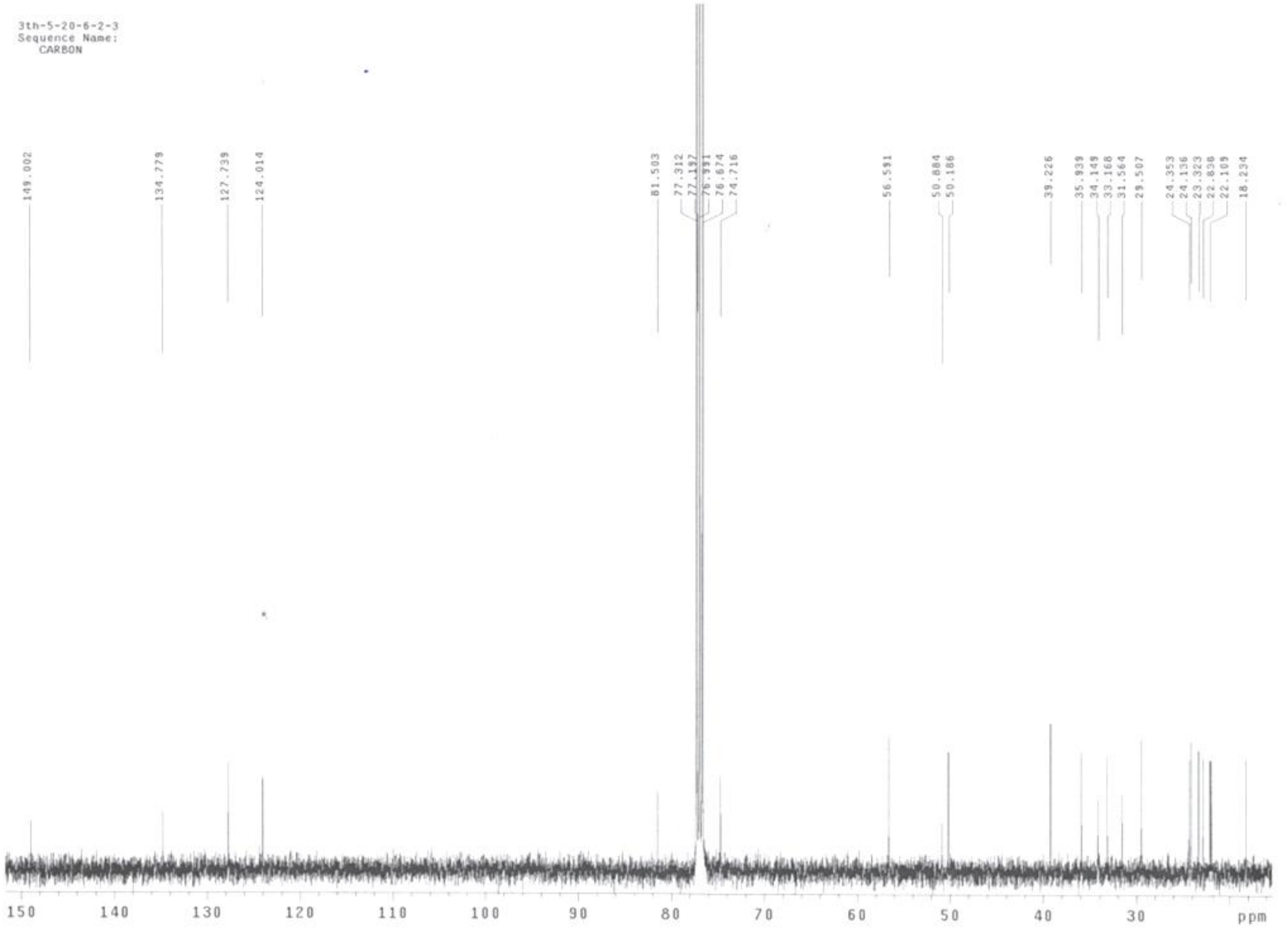

Figure S12. DEPT spectrum (100 MHz) of compound **2** in $CDCl_3$.

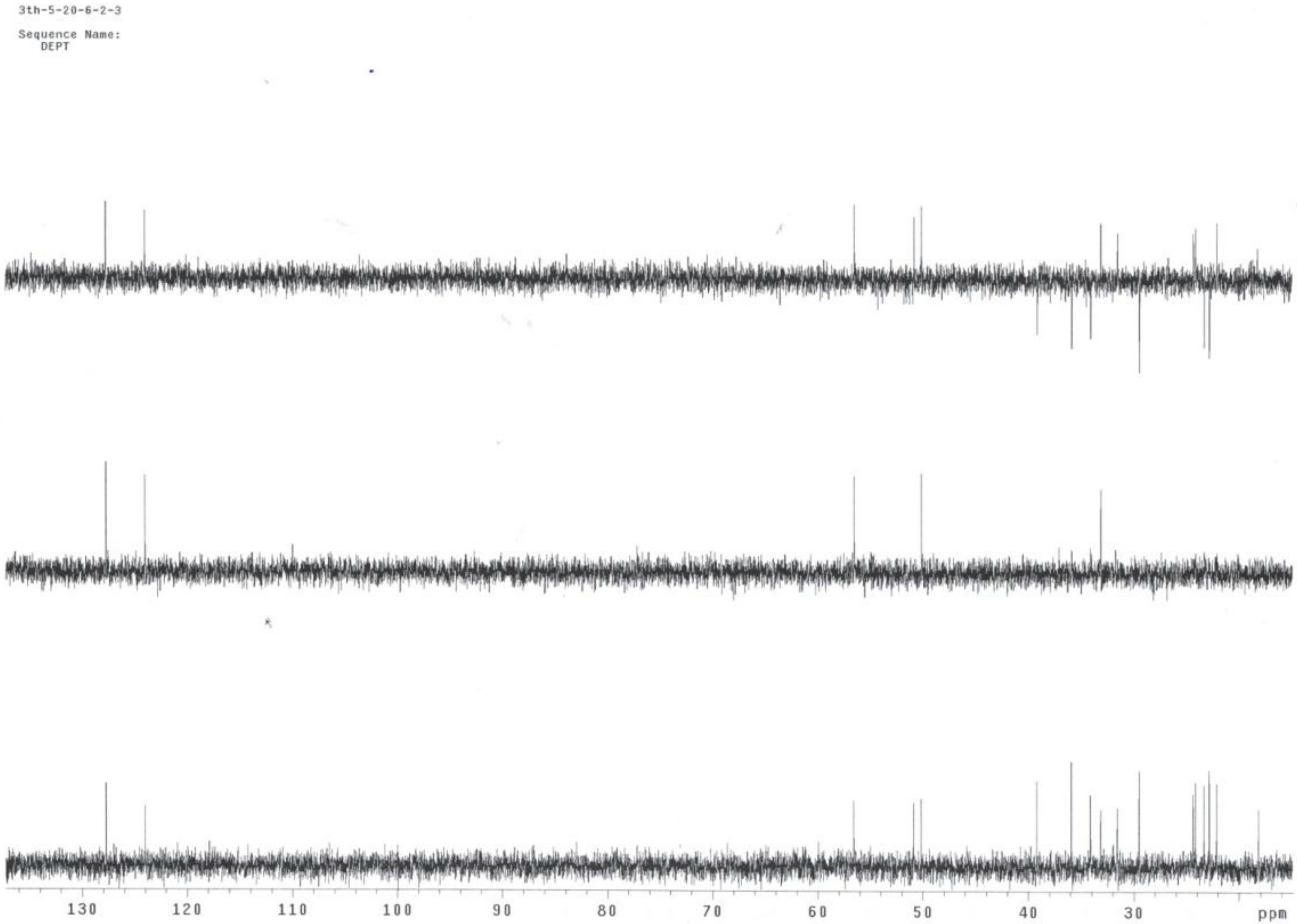

Figure S13. HSQC spectrum (400 MHz) of compound **2** in $CDCl_3$.

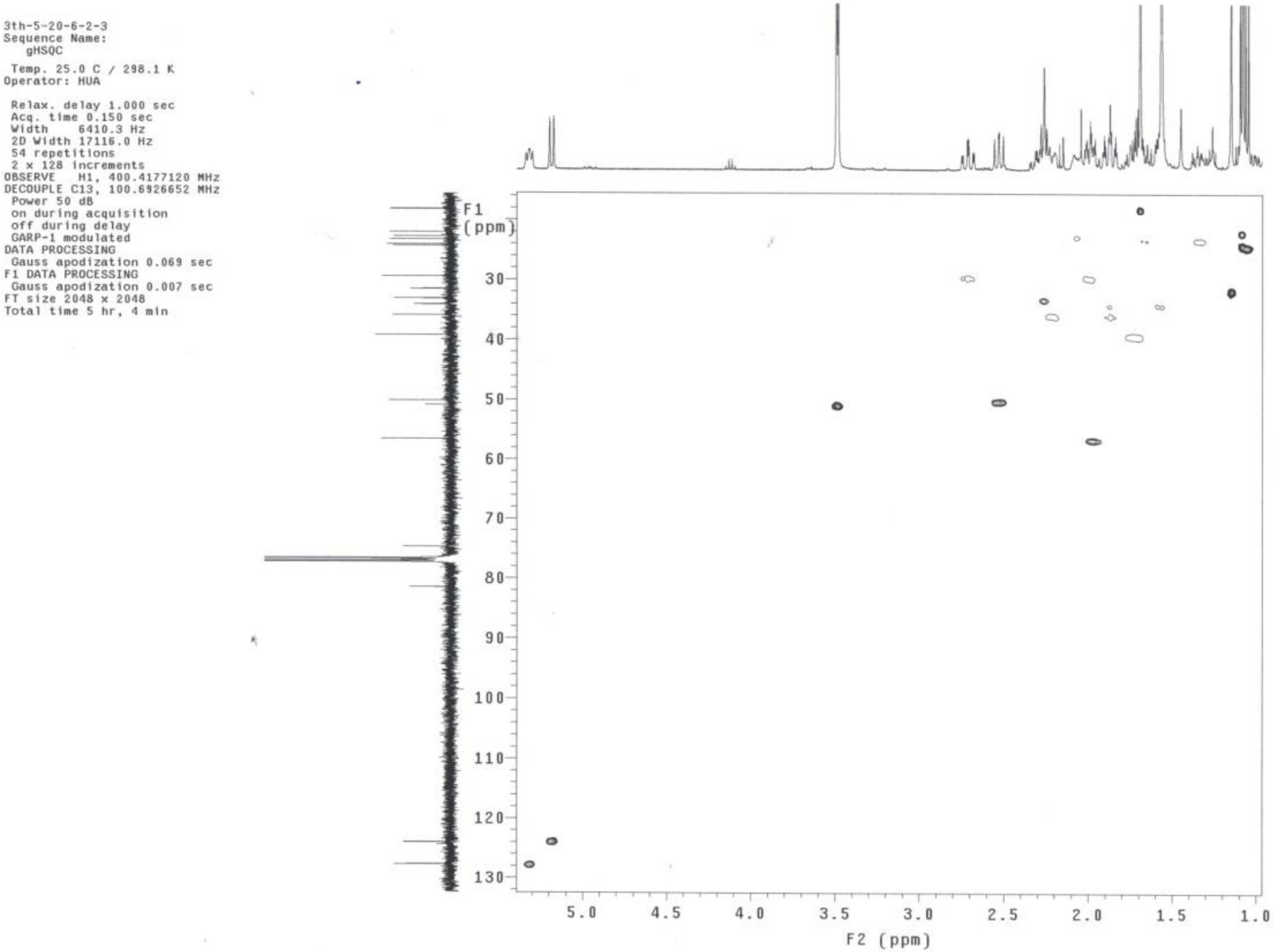

Figure S14. HMBC spectrum (400 MHz) of compound **2** in $CDCl_3$.

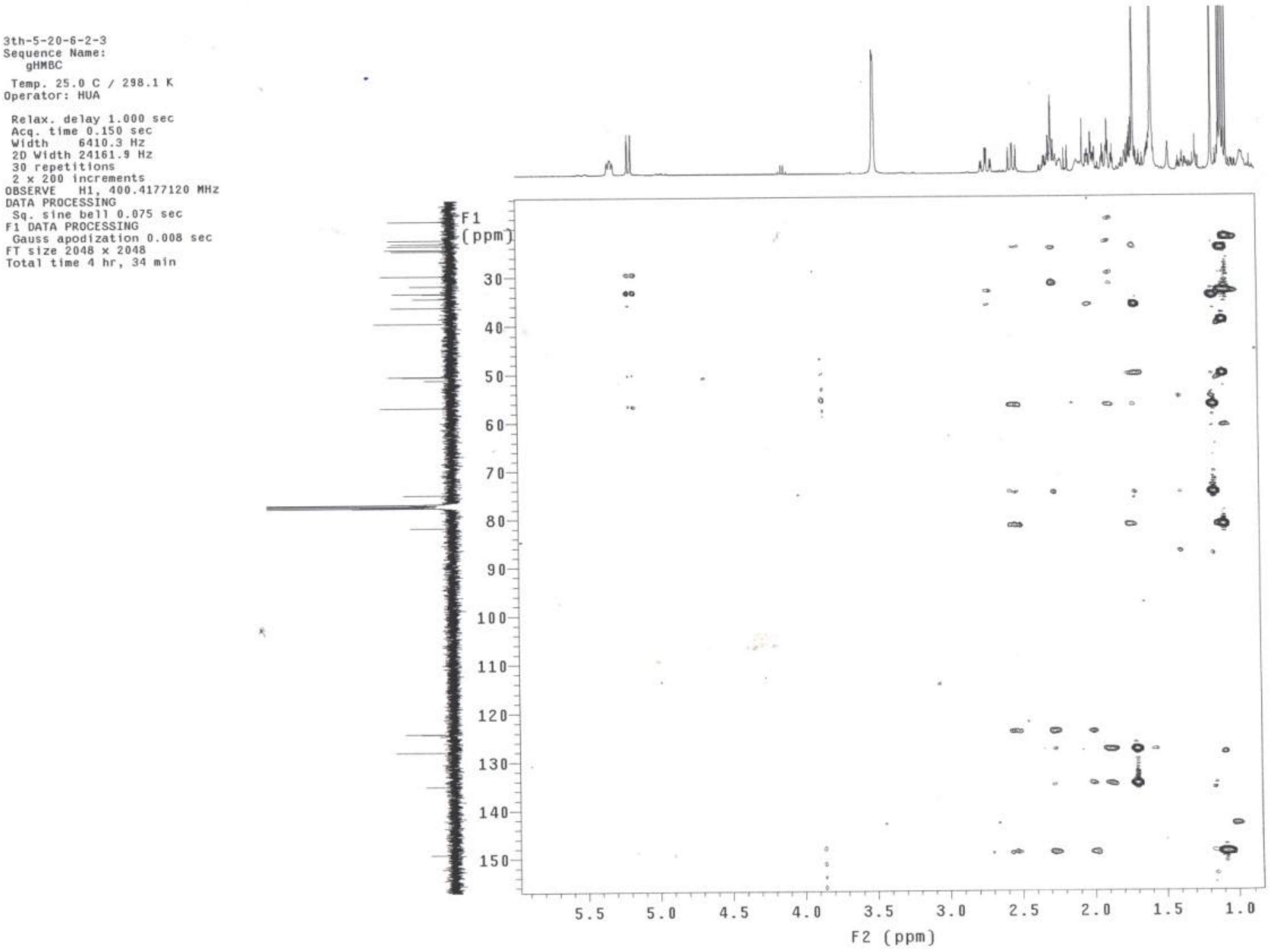

Figure S15. COSY spectrum (400 MHz) of compound **2** in $CDCl_3$.

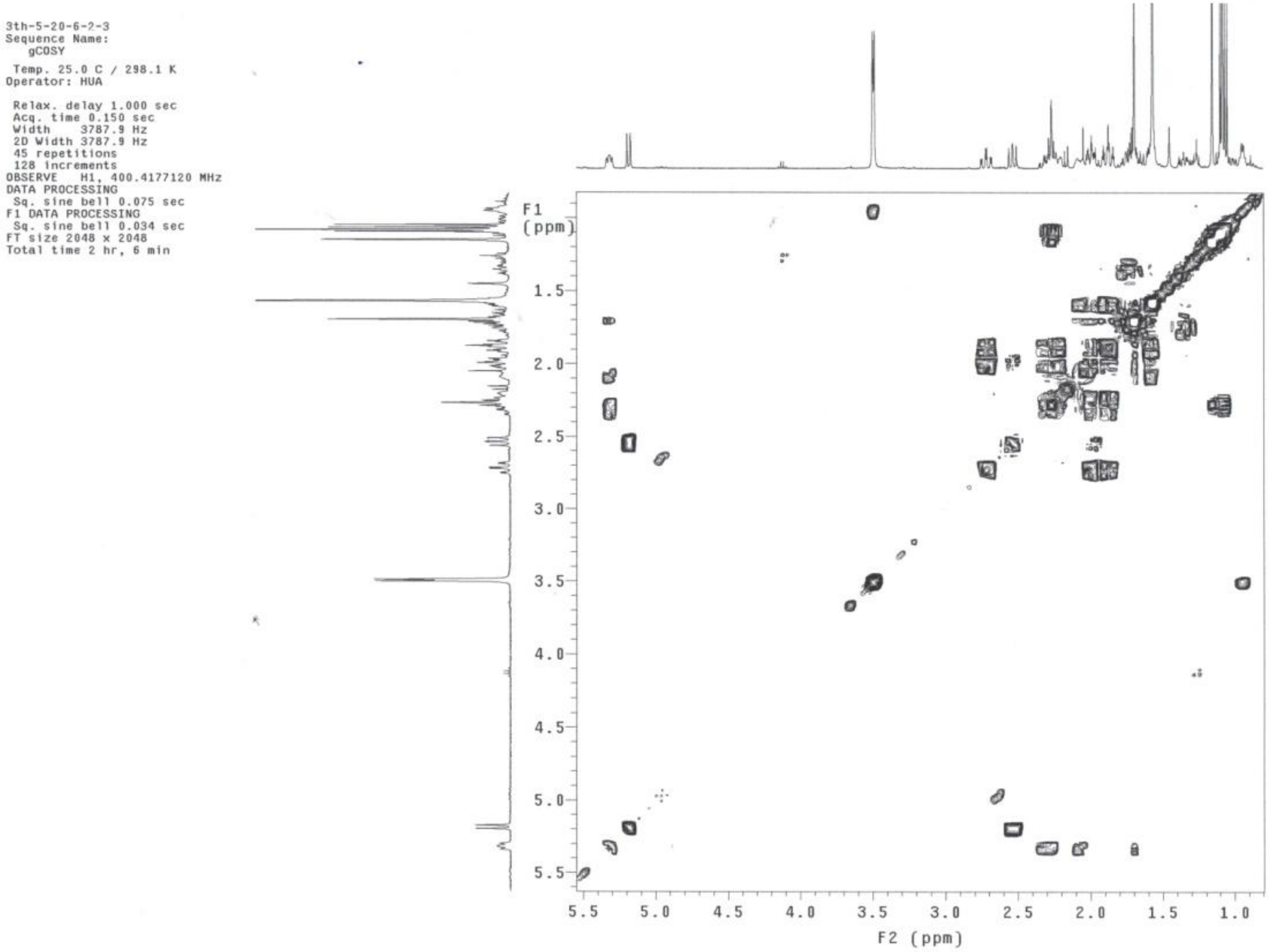

Figure S16. NOESY spectrum (400 MHz) of compound **2** in $CDCl_3$.

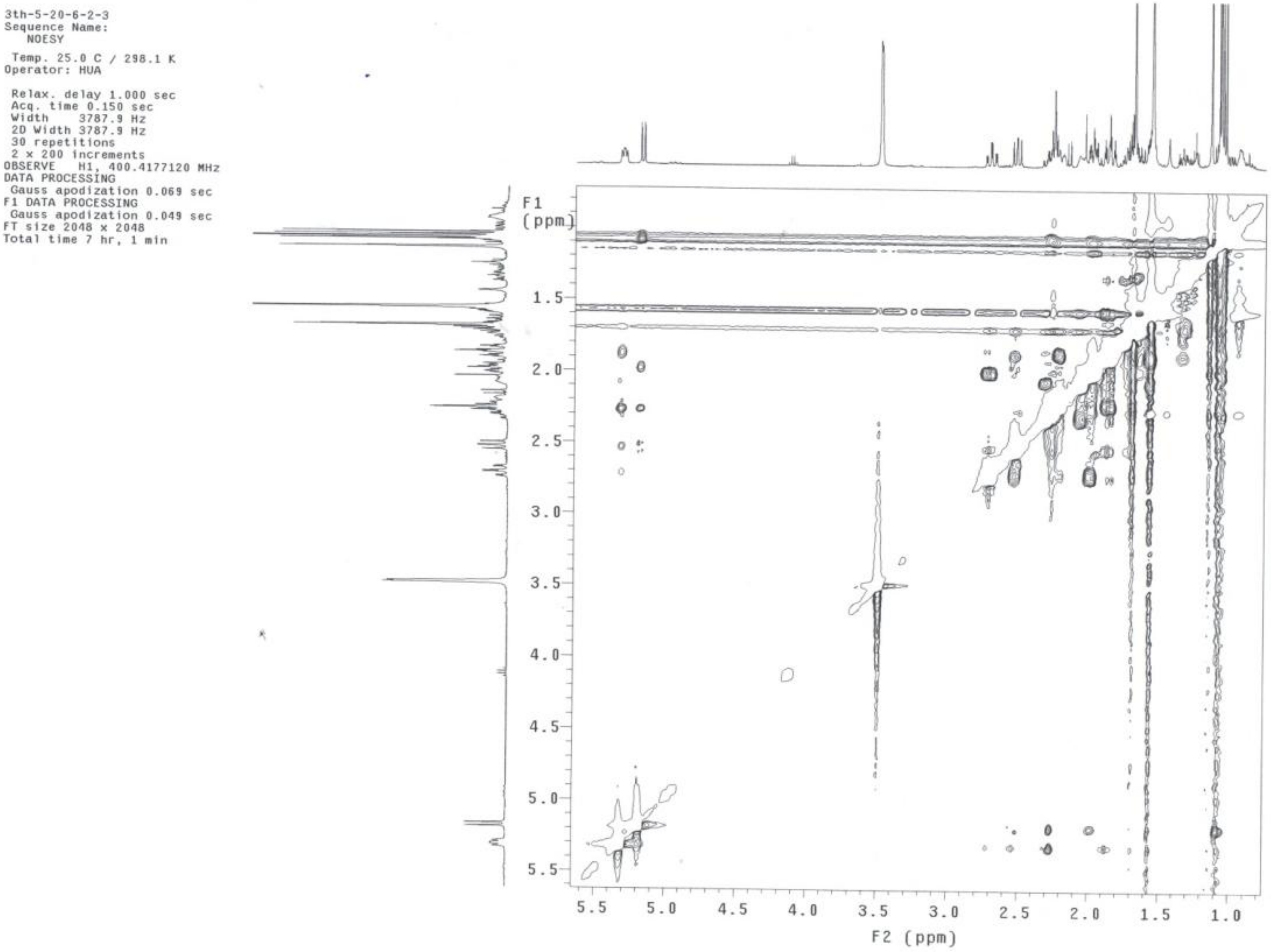

Supplement: Supplementary File 1 — Supplementary Information (PDF, 939 KB) [file marinedrugs-12-00385-s001.pdf]
